# Octopamine signaling in the metazoan pathogen *Schistosoma mansoni*: localization, small-molecule screening and opportunities for drug development

**DOI:** 10.1242/dmm.033563

**Published:** 2018-07-30

**Authors:** Nelly El-Sakkary, Steven Chen, Michelle R. Arkin, Conor R. Caffrey, Paula Ribeiro

**Affiliations:** 1 Institute of Parasitology, McGill University, Macdonald Campus, 21, 111 Lakeshore Road, Ste Anne de Bellevue, Quebec, Canada H9X-3V9; 2 Small Molecule Discovery Center, Department of Pharmaceutical Chemistry, University of California San Francisco, San Francisco, CA 94158, USA; 3 Center for Discovery and Innovation in Parasitic Diseases, Department of Pathology, University of California San Francisco, San Francisco, CA 94158, USA

**Keywords:** Schistosoma mansoni, Synapsin, Nervous system, Biogenic amine, Neuromuscular, Octopamine, Dopamine, Drug discovery

## Abstract

Schistosomiasis is a tropical disease caused by a flatworm trematode parasite that infects over 200 million people worldwide. Treatment and control of the disease rely on just one drug, praziquantel. The possibility of drug resistance coupled with praziquantel's variable efficacy encourages the identification of new drugs and drug targets. Disruption of neuromuscular homeostasis in parasitic worms is a validated strategy for drug development. In schistosomes, however, much remains to be understood about the organization of the nervous system, its component neurotransmitters and potential for drug discovery. Using synapsin as a neuronal marker, we map the central and peripheral nervous systems in the *Schistosoma mansoni* adult and schistosomulum (post-infective larva). We discover the widespread presence of octopamine (OA), a tyrosine-derived and invertebrate-specific neurotransmitter involved in neuromuscular coordination. OA labeling facilitated the discovery of two pairs of ganglia in the brain of the adult schistosome, rather than the one pair thus far reported for this and other trematodes. In quantitative phenotypic assays, OA and the structurally related tyrosine-derived phenolamine and catecholamine neurotransmitters differentially modulated schistosomulum motility and length*.* Similarly, from a screen of 28 drug agonists and antagonists of tyrosine-derivative signaling, certain drugs that act on OA and dopamine receptors induced robust and sometimes complex concentration-dependent effects on schistosome motility and length; in some cases, these effects occurred at concentrations achievable *in vivo*. The present data advance our knowledge of the organization of the nervous system in this globally important pathogen and identify a number of drugs that interfere with tyrosine-derivative signaling, one or more of which might provide the basis for a new chemotherapeutic approach to treat schistosomiasis.

This article has an associated First Person interview with the first author of the paper.

## Abstract (precis)

**Summary:** The nervous system of *Schistosoma mansoni* has been mapped and the widespread presence of octopamine revealed. The identification of agonists and antagonists of tyrosine-derivative signaling might provide opportunities for drug repurposing.

## INTRODUCTION

The blood fluke *Schistosoma mansoni* (Phylum Platyhelminthes, Class Trematoda) is one of the causative agents of the ‘neglected’ tropical disease (NTD), schistosomiasis. Worldwide, more than 200 million people have this disease, with almost 800 million at risk of *Schistosoma* infection, and transmission has been reported in 78 countries in Africa, South America, the Middle East, Caribbean and parts of China and South-East Asia (http://www.who.int/news-room/fact-sheets/detail/schistosomiasis; Steinmann et al., 2006). Disease morbidity, owing to inflammation and fibrosis associated with the parasite's eggs, is typically chronic and can be painful and debilitating (Colley et al., 2014), hampering both personal productivity and community development.

A single drug, praziquantel (PZQ), has been used to treat and control schistosomiasis since the early 1980s. However, PZQ has a number of pharmacological and pharmaceutical problems (e.g. incomplete efficacy profile at the high 40 mg/kg dose, poor pharmacokinetics and unpalatable taste), which undermine efforts to eliminate the disease (Andrews et al., 1983; Sabah et al., 1986; Meyer et al., 2009; Caffrey, 2007, 2015). Additionally, there are concerns over the possible development of drug resistance (Melman et al., 2009; Doenhoff et al., 2008), particularly given the recent increased international efforts to expand the distribution of PZQ (The London Declaration on NTDs, 2012: http://partnerships.ifpma.org/partnership/the-london-declaration-on-ntds; The WHO Roadmap for Implementation, 2012: www.who.int/neglected_diseases/NTD_RoadMap_2012_Fullversion.pdf).

The increasing reliance, therefore, on a single partially effective drug highlights the importance of discovering new anti-schistosomal chemistries. In parasitic helminths (nematodes and flatworms), the nervous system has been a rich source of drug targets (Robertson and Martin, 2007; Kaminsky et al., 2008; Gutman et al., 2010; Wolstenholme, 2011) with many anthelmintics acting on proteins involved in neuronal signaling to induce spastic or flaccid paralysis (Bueding et al., 1972; Bloom, 1981; Geerts et al., 1989; Gill et al., 1991; Geary et al., 1993; Caffrey et al., 2012), and subsequent elimination of the parasite from the host.

The schistosome nervous system underpins successful migration of the parasite through the host (Crabtree and Wilson, 1980), feeding and egg laying (Maule et al., 2005). The organization of the nervous system has been primarily inferred by comparisons with other flatworms, including trematodes (Fairweather et al., 1988; Skuce et al., 1990; Halton et al., 1991; Maule et al., 1992; Brownlee et al., 1994), in addition to a small number of studies on *S. mansoni* adults (Bennett and Bueding, 1971; Gustafsson, 1987) and cercariae (infective larvae; Cousin and Dorsey, 1991; Collins et al., 2011). Apart from these studies, reports on nervous system components have focused on the identification of neurotransmitters (Solis Soto and De Jong Brink, 1994; El-Shehabi et al., 2012), rather than descriptions of its overall organization. Previous immunolocalization studies in trematodes indicate that the central nervous system (CNS) comprises a brain [cerebral ganglia (CG)], a bi-lobed structure made up of a dense axon-rich neuropile that is connected by a ring commissure (Halton and Maule, 2004). Pairs of dorsal, ventral and lateral nerve chords (NCs) extend from each lobe of the CG (Cousin and Dorsey, 1991). These longitudinal NCs are cross-linked with transverse commissures along the length of the worm, providing an orthogonal, or ladder-like, pattern (Hyman, 1951; Halton and Maule, 2004; Collins et al., 2011).

Trematodes also have a peripheral nervous system (PNS) made up of finer nerve fibers and plexuses. These connect to all major body structures including the somatic musculature, the tegument (surface), the oral and ventral suckers, the reproductive organs and the alimentary tract (Halton and Gustafsson, 1996; Halton and Maule, 2004). In *S. mansoni*, numerous nerve endings and innervated papillae have been identified in the tegument, where they are presumed to participate in sensation in cercariae (Gordon et al., 1934; Dorsey and Stirewalt, 1971; Nuttman, 1971; Short and Kuntz, 1976; Cousin and Dorsey, 1991) and adult parasites (Gordon et al., 1934; Smith et al., 1969).

The schistosome nervous system is modulated by both peptidergic (Gustafsson, 1987; Humphries et al., 2004; Ribeiro and Geary, 2009) and small classical transmitters (Gustafsson, 1987; Day et al., 1994; Ribeiro and Geary, 2009; El-Shehabi and Ribeiro, 2010; McVeigh et al., 2011; El-Shehabi et al., 2012; Patocka et al., 2014). Among the classical transmitters are acetylcholine (ACh), glutamate and the biogenic amines (BAs). BAs are derived from the aromatic amino acids, tyrosine and tryptophan (Koslow and Butler, 1977; Sainio et al., 1996), or histidine (Schayer, 1960). BAs represent the largest subset of classical transmitters in the animal kingdom (Maule et al., 2005) and contribute to schistosome motility (Pax et al., 1984; Ercoli et al., 1985; Day et al., 1994; El-Shehabi et al., 2012; Patocka et al., 2014).

Among the invertebrate-specific BAs are octopamine (OA) and its precursor tyramine (TA), which are metabolically derived from tyrosine. In locusts (Dougan and Wade, 1978) and molluscs (Burrows and Pfluger, 1995), OA and TA are involved in motor control (Hoyle, 1985; Mulloney et al., 1987; Roeder et al., 1998; Verlinden et al., 2010). Apart from OA and TA signaling, adrenergic signaling can co-exist in some invertebrates, although it was lost from nematodes and most arthropods (Bauknecht and Jékely, 2017). In some arthropods, OA signaling can perform several functions fulfilled by adrenaline and noradrenaline in vertebrates (Evans et al., 1976; Goosey and Candy, 1980; Evans, 1981). OA and TA are also present in mammals, but only in trace amounts (Borowsky et al., 2001): this differential affords the opportunity to pursue chemical control of invertebrates (Evans and Gee, 1980; Dudai, 1982; Roeder et al., 1998). The presence of an OA-based neurotransmission system in the schistosome is unknown; however, if identified, a better understanding of that system could prove useful for the development of new drugs.

In this context, we first determined the organization of the CNS and PNS using the neuronal marker synapsin in two developmental stages of *S. mansoni* that parasitize the human: namely, adults and post-infective larvae (schistosomula). We discovered that OA is widely distributed in neurons of the peripheral nerve net that innervate muscle, suggesting a contribution to neuromuscular control. Using schistosomula, we then performed quantitative whole-organism analyses to measure the effects of OA, related tyrosine derivatives and synthetic neuromodulatory drugs on parasite length and motility.

## RESULTS

### Labeling with the neuronal marker synapsin reveals the organization of the nervous system in the parasitic stages of *S. mansoni*

The nervous system of post-infective schistosomula and adult *S. mansoni* was labeled using a monoclonal antibody to *Drosophila* synapsin, which had been previously shown to recognize synapsin in *S. mansoni* invasive larvae known as cercariae (Collins et al., 2011). Samples were treated with a signal amplification reagent (Tyramide Signal Amplification, TSA), as described (Collins et al., 2011), to increase the sensitivity of the assay. Immunolabeling without the TSA amplification reagent did not yield a detectable signal.

**Fig. 1.**
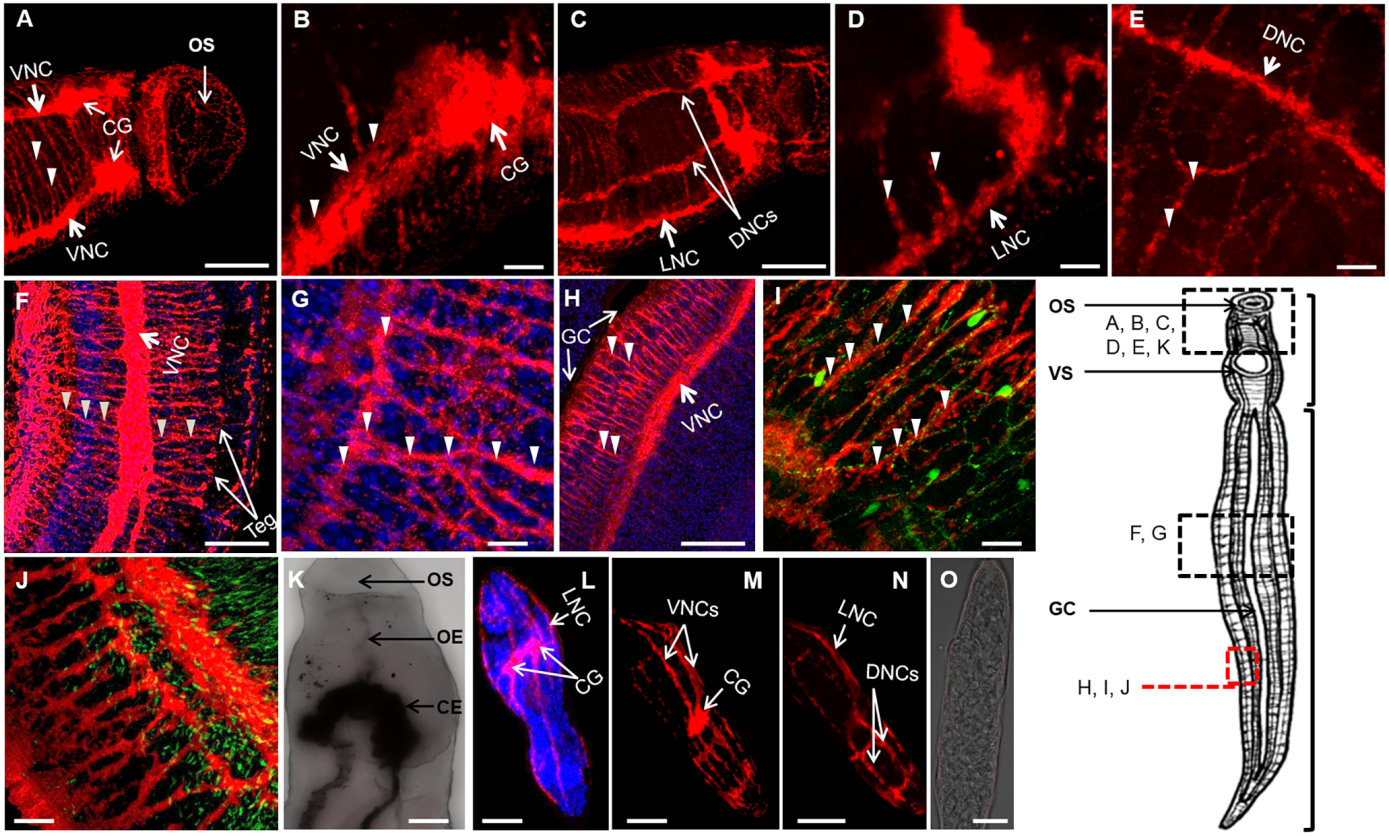
**Architecture of the schistosome nervous system.** (A) The neuronal marker synapsin was labeled with a monoclonal anti-synapsin antibody (anti-SYNORF1) and a secondary antibody conjugated to HRP followed by tyramide signal amplification, which produces a red color. Arrowheads point to nerve chords, nerve fibers or nerve cell bodies. The position of the worm in each image is approximated relative to the scheme of the worm's body on the right in which the locations of the oral sucker (OS), ventral sucker (VS), gynecophoral canal (GC), the head (upper bracket) and body (lower bracket) are also indicated. (B) The ventral nerve chords (VNCs), joined by transverse commissures, and cerebral ganglia (CG) are visible. (C,D) Lateral nerve chords (LNCs) are visible on either side of the worm. (C,E) The dorsal nerve chords (DNCs) are also observed. (F,G) The fine PNS nerve fibers are visible throughout the body and extend to the surface [tegument (Teg), indicated with arrows in F] and intersect with the main longitudinal nerve chords. (H) Short nerve fibers of the PNS extend from the VNCs to the GC flap, as indicated with arrows. (I) The neurotransmitter 5HT (green), was co-immunolabeled with anti-synapsin and the labeling pattern shows a close juxtaposition of nerve fibers. (J) Actin (green) was co-immunolabeled with synapsin (red) and is apparent along the main nerve chords of the CNS. Incubation of worms with secondary antibody and amplification reagent alone did not yield significant labeling, as demonstrated by the negative control image that was overlaid with the brightfield image (K): the oesophagus (OE) and caecum (CE) are indicated. Scale bars: 100 μm at 20× magnification in panels A, C, F, H and K; 20 μm at 63× magnification in panels B, D, E, G, I, J, L-O. (L,M) In D7 schistosomula, synapsin was labeled in the CG and VNCs that projected longitudinally. (L,N) DNCs and LNCs are also labeled. (O) Incubation with the amplification reagent and secondary antibody alone did not yield significant labeling, as demonstrated with the transmitted image overlay.

In the adult schistosome, synapsin is distributed throughout the CNS and PNS (Fig. 1A-H, Movies 1-3). The bi-lobed brain, characteristic of flatworms, and the commissural ring linking each ganglion are labeled just posterior of the oral sucker (Fig. 1A). Three pairs of longitudinal NCs originate from the CG: ventral (Fig. 1A,B), lateral (Fig. 1C,D) and dorsal (Fig. 1C,E). These chords are cross-linked by synapsin-positive transverse commissures along the length of the worm body (Fig. 1A). PNS nerve fibers intersecting the longitudinal nerve fibers of the CNS are labeled with synapsin (Fig. 1F). In the mid-body, a ventral NC is visible along one flap of the gynecophoral canal (a ventral groove along the long axis of the male worm that holds the cylindrical female worm; Fig. 1H). The gynecophoral canal flaps are highly innervated, and sensation and control of the flaps are probably important in the coupling of male and female worms. Co-staining with DAPI reveals that synapsin labels what appear to be neuronal cell bodies at the junctions between a longitudinal NC and connecting perpendicular fibers. These fibers might belong to the PNS or are orthogonal nerve fibers of the CNS (Fig. 1G). The gynecophoral canal flaps are highly innervated, and sensation and control of the flaps are likely important in the coupling of male and female worms (Fig. 1H).

To compare the distribution of synapsin with that of a known neurotransmitter, we co-labeled adult worms using antibodies against synapsin (red) and serotonin [5-hydroxytryptophan (5HT); green; Fig. 1I]. Labeling of 5HT is close to that of synapsin with some overlap, although a perfect colocalization was not observed. This labeling suggests that 5HT and synapsin are enriched in separate regions of the neuron or that synapsin is not as abundant in schistosome serotonergic neurons as we observed elsewhere in the parasite.

To determine an association of neurons with muscle, we co-labeled adult worms with synapsin-specific antibody (red) and fluorescein isothiocyanate (FITC)-conjugated phalloidin to label actin (green; Fig. 1J). Actin is visible as punctate regions close to and overlapping with neurons, indicating a close association between muscle and the nervous system. Negative controls that employed secondary antibody and detection reagent without primary antibody did not produce significant labeling, as shown in the overlay with the brightfield image (Fig. 1K).

Synapsin also labeled the CNS of fixed 7-day-old (D7) schistosomula. Labeling of the PNS was not noted, perhaps suggesting that the finer nerve fibers of the PNS are not detectable under the conditions used. A single pair of CG, similar to that identified in cercariae (Cousin and Dorsey, 1991; Collins et al., 2011), is strongly labeled in the center of the schistosomulum's body (Fig. 1L,M). Ventral NCs extend longitudinally from the CG (Fig. 1L,M), and lateral (Fig. 1L,N) and dorsal NCs (Fig. 1N) are also visible. Together, the NCs with the transverse commissures produce a cage-like structure in the schistosomulum (Fig. 1M,N). The DAPI counterstain reveals the densely nucleated schistosomulum (Fig. 1L). Labeling schistosomula with the secondary antibody and the TSA reagent without primary antibody was negative (Fig. 1O). Overall, we conclude that synapsin is a useful neuronal marker in adults and schistosomula of *S. mansoni*.

### Labeling with specific antibody reveals the widespread presence of OA in the CNS and PNS of *S. mansoni* adults and schistosomula

Adult males and females, and schistosomula, were fixed, permeabilized and probed with anti-OA antibody, followed by an Alexa-488 (green)-conjugated secondary antibody. DAPI was used as a counterstain in some experiments and the neuronal marker synapsin was used to verify localization to nervous tissue.

**Fig. 2.**
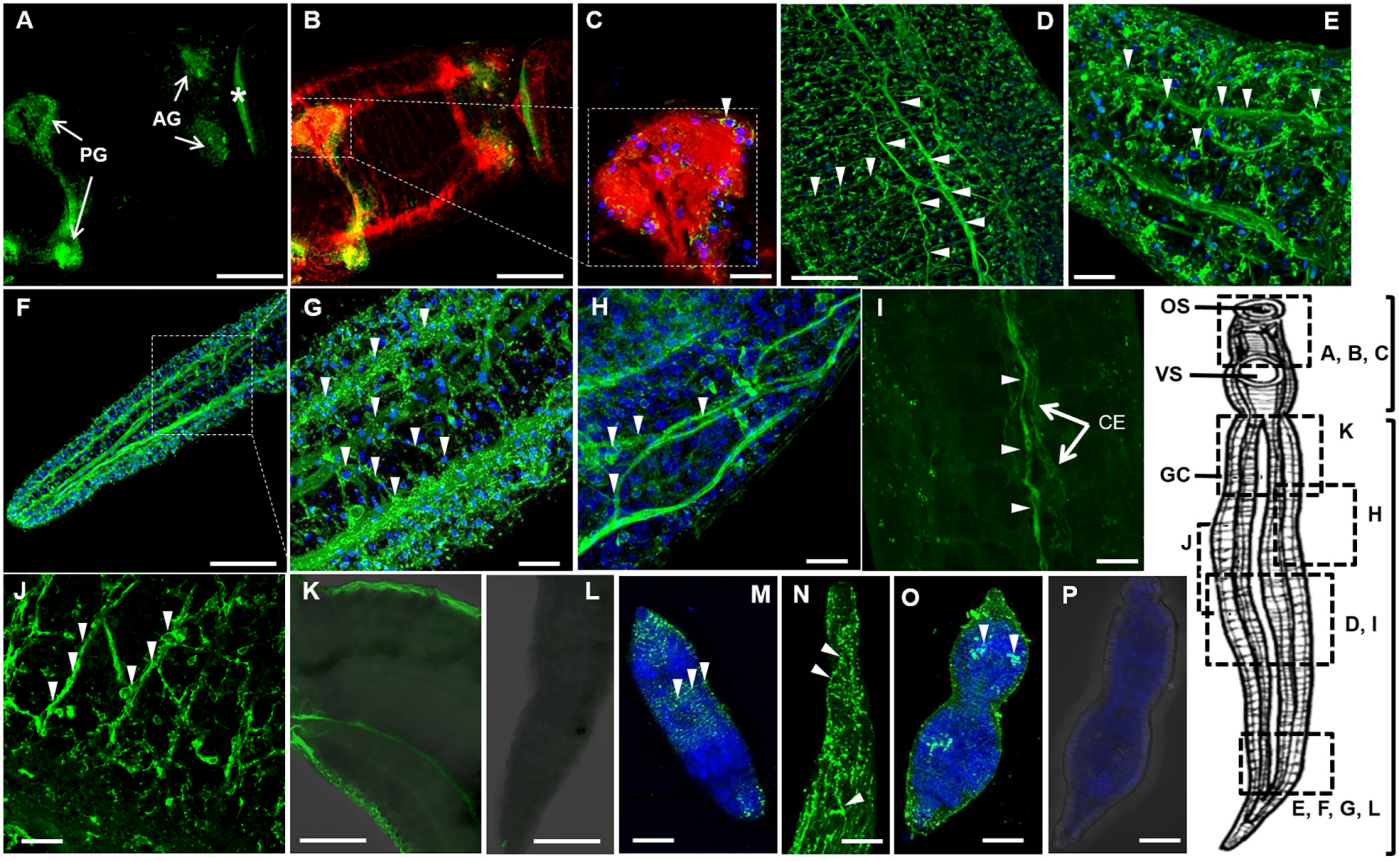
**OA is found in the central and peripheral nervous systems.** OA was labeled with an OA-specific primary antibody and an Alexa-488 (green)-conjugated secondary antibody; DAPI was used as a counterstain (blue). Arrowheads indicate nerve chords, nerve fibers or cell bodies. (A) OA is localized to anterior ganglia (AG) and posterior ganglia (PG) in the head of the adult male worm, a finding that is supported by synapsin co-labeling (B,C; in red). The AG are located posterior of the oral sucker, whereas the PG are anterior of the ventral sucker. Non-specific surface labeling of OA in (A) is indicated with an asterisk. (D-H) OA is found in the longitudinal nerve chords of the CNS and PNS in both males (D,E) and females (F-H). (E,G,H) At higher magnification, OA is identified in cell bodies. (I) An octopaminergic neuron lines the parasite's caecum (CE), indicated by arrowheads. (J) OA is present in what appears to be the submuscular peripheral nerve net of the PNS. (K) Worms were also probed with anti-OA antibody pre-adsorbed with OA or (L) secondary antibody alone: fluorescence, if noted, was non-specific. OA is also detected in D7 schistosomula, indicated by labeling with Alexa-488-conjugated antibody. Innervation of the PNS (M,N) and possibly of the CG (O) is visible. (P) The secondary antibody alone did not yield non-specific labeling. Scale bars: 100 μm at low magnification (20×) in panels A, B, D, F, K and L; 20 μm at high magnification (63×) in panels C, E, G, H-J, M-P.

Strikingly, OA strongly localized to what appeared to be two pairs of ganglia in the parasite's head. One pair is positioned just posterior of the oral sucker (Fig. 2A, right) and the second is anterior of the ventral sucker (Fig. 2A, left). The presence of two pairs of ganglia was confirmed by the synapsin counterstain (Fig. 2B) and is a new finding for trematodes. We term these anterior ganglia (AG) and posterior ganglia (PG). OA is also present in the commissural ring, which laterally joins the lobes of the PG (Fig. 2A). The localization of OA to nervous tissue was confirmed by generating a composite image of OA and synapsin labeling that shows regions of yellow (Fig. 2B). At a higher magnification (63×), we observed what appear to be cell bodies containing OA along the surface of a ganglion neuropile (Fig. 2C).

Apart from the two pairs of brain ganglia, OA is present in the longitudinal NCs and transverse commissures of the CNS along the length of the body, as well as in fine nerve fibers of the PNS that extend to the worm surface in males (Fig. 2D,E) and females (Fig. 2F-H). The labeling pattern of OA is similar to that of synapsin, confirming that OA localizes to the nervous system. OA is detected along a large nerve fiber running along the caecum, which could suggest a function in peristalsis during feeding and digestion (Fig. 2I). In the male, at 63× magnification, OA is strongly labeled in a network of fibers resembling the peripheral submuscular nerve net (Reuter and Gustafsson, 1995) of the PNS with criss-crossing fibers innervating the body-wall muscle (Fig. 2J). This localization suggests that OA contributes to neuromuscular control (motility). Negative controls employing anti-OA antibody pre-adsorbed with OA (Fig. 2K) yielded a non-specific fluorescence on the surface of the worm, unlike the labeling patterns described above. Controls employing secondary antibody alone were negative (Fig. 2L).

Unlike adult parasites, OA is largely restricted to the PNS in D7 schistosomula. Diffuse labeling is present in concentric circles near the surface of the animal (Fig. 2M). This pattern is similar to that previously described for other neuronal proteins in this larval stage (Patocka et al., 2014). OA is also observed in fine nerve fibers and in what appear to be cell bodies, which are connected in a web-like organization along the length of the body directly beneath the surface (Fig. 2N). Thus, the localization of OA is more disorganized in D7 somules than in adult schistosomes, consistent with a nervous system still undergoing development. Labeling of a pair of punctate sites in the upper half of the schistosomula might indicate the presence of OA in the developing CG (Fig. 2O). Labeling with secondary antibody alone was negative (Fig. 2P). Overall, the results demonstrate that OA is present in the PNS and in the developing CG of D7 schistosomula.

### OA is found in the female ovary and in the nerve net of the developing embryo

**Fig. 3.**
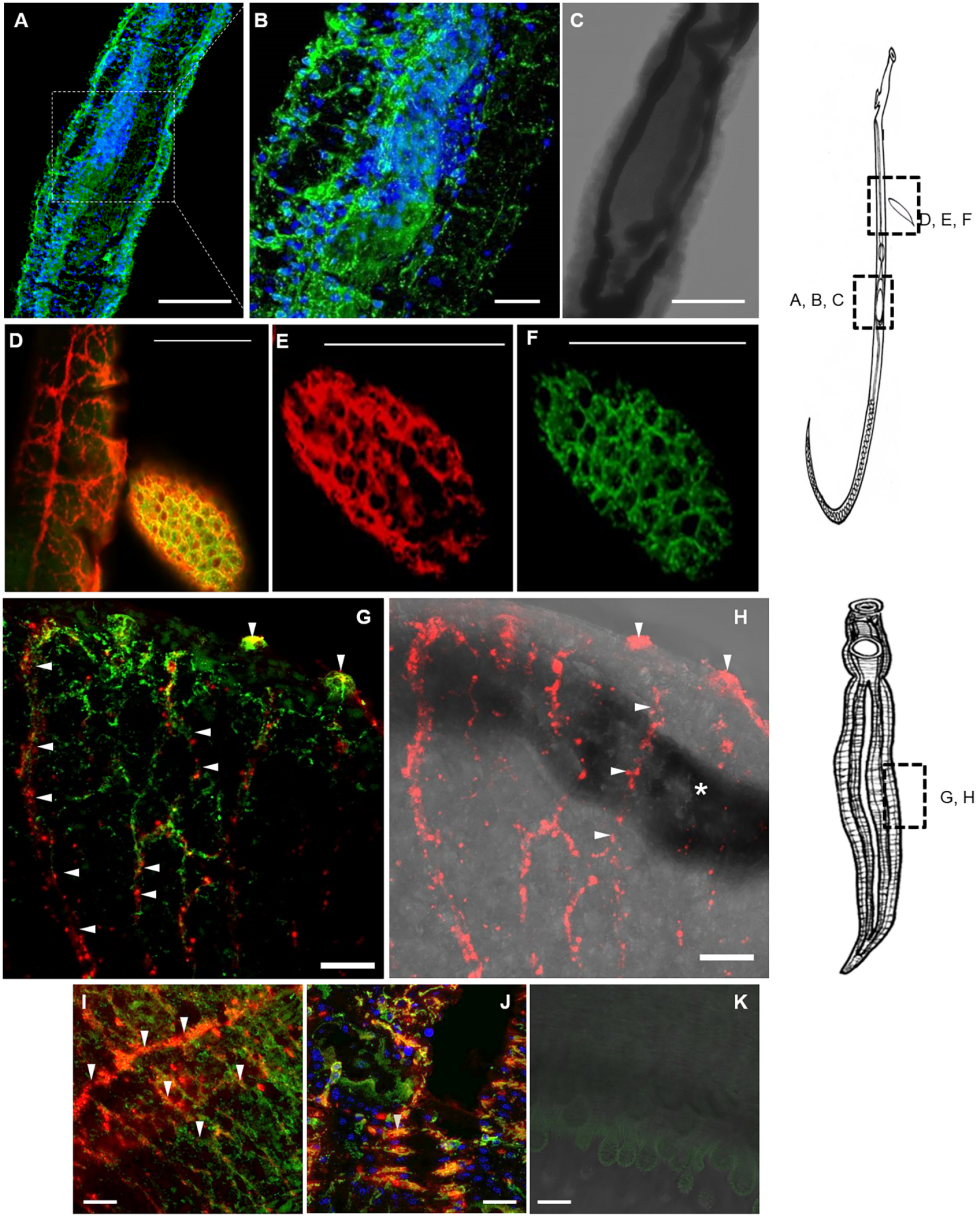
**OA and synapsin are found in the ovary of the female, in the developing embryo and innervating the surface of the worm.** The region in the adult female worm or adjacent egg is indicated in the diagram of the female worm on the right. OA was labeled with an anti-OA primary antibody and an Alexa-488 (green)-conjugated secondary antibody. (A,B) Octopaminergic neurons run along the length of the ovary, coinciding with a concentration of nuclei (Neves et al., 2005). (C) The ovary is also shown in the brightfield image. Colocalization of OA with (D) anti-synapsin (yellow) and either (E) anti-synapsin (red) or (F) anti-OA (green) alone reveals the organization of the nerve fibers in the developing embryo. For the adult male worm, regions in the panels are indicated in the lower diagram to the right of the image grid. In the male, OA and synapsin are colocalized in the surface tubercles (G) with synapsin alone in the synapsin-transmitted light image (H). The asterisk in H indicates a dark line running from left to right in the brightfield image overlay, which is due to a female worm within the male's gynecophoral canal just below the plane of view. (I) Synapsin appears to be enriched in CNS nerve fibers and OA in PNS nerve fibers. (J) The surface of the gynecophoral canal is innervated by a cluster of short nerve fibers containing both OA and synapsin, as indicated by the labeling in yellow. Scale bars: 100 μm with 20× magnification in panels A, C-F; 20 μm at high magnification (63×) in panels B, G-K.

OA is prominent in the female reproductive tract (Fig. 3A), including the ovary (Fig. 3B,C), where we observed a concentration of DAPI-stained nuclei that belong to the oocytes, as previously described (Neves et al., 2005). A schistosome egg co-labeled with anti-OA and anti-synapsin antibodies is shown adjacent to a female worm. The embryo within the egg shows the apparent colocalization of OA and synapsin (Fig. 3D-F), as indicated by the mesh-like pattern of intense yellow fluorescence. Although the general organization of the embryo's nervous system is known, as determined by staining with Hematoxylin and Eosin or Giemsa (Jurberg et al., 2009), little is known about neurotransmitters or the organization of neurotransmitter-specific neurons in the embryo. The size of the embryo (>100 µm in length), its elongated shape and its occupation of most of the internal area of the egg (Jurberg et al., 2009) suggests that it is in a later stage of development, possibly at the seventh of the eight stages originally defined (Jurberg et al., 2009). To conclude, OA is widely distributed in the nervous system of the female reproductive tract and embryo.

### OA innervates the surface of adult parasites

We identified OA in neurons close to the surface of adult worms, including in surface tubercles of males (Fig. 3G) which are enriched in sensory nerve fibers and nerve endings (Kruger et al., 1986; Macdonald et al., 2014). Colocalization of OA and synapsin in the tubercles confirms the presence of OA in neuronal tissue (Fig. 3G). The innervation of tubercles connecting to the subtegumental nerve net suggests a function in sensation for OA. Also, the progression of nerves that contain OA from the tubercles into the body and their intersection with synapsin-labeled longitudinal NCs of the CNS indicate a possible efferent innervation of octopaminergic sensory neurons. Labeling of synapsin from deep in the worm body to the tubercles is evident with the synapsin-transmitted light image overlay (Fig. 3H).

A merging of the octopaminergic neurons of the PNS with synapsin-labeled neurons in the CNS is evident in the mid-body (Fig. 3I). OA and synapsin also show a close juxtaposition along the outer flaps of the male worm's gynecophoral canal, indicating extensive innervation (Fig. 3J). Controls using secondary antibody and the amplification reagent without primary antibody were negative (Fig. 3K).

### Biogenic amines, including tyrosine derivatives, modulate schistosomulum length and movement

**Fig. 4.**
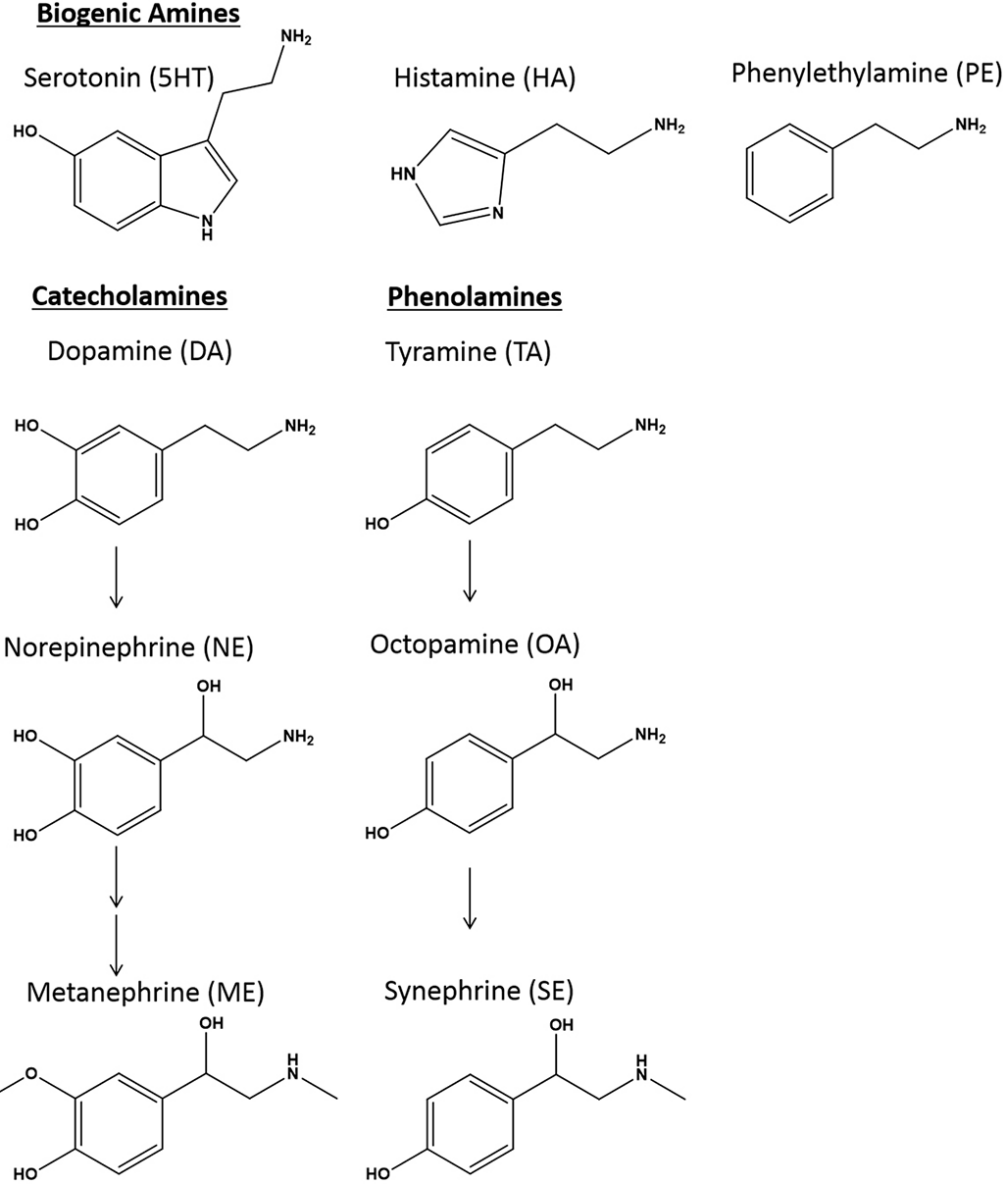
**Biogenic amine structures.** The indicated BAs are derived from histidine [histamine (HA)] or aromatic amino acids such as tyrosine and tryptophan. Structurally related tyrosine derivatives include the catecholamines (DA, NE and ME) and the phenolamines (TA, OA and SE).

The widespread occurrence of OA in the schistosome CNS and PNS suggests that OA is important in neuromuscular control. To understand whether exogenously added tyrosine-derived neurotransmitters could influence motor control, we phenotypically measured D7 schistosomula for changes in motility and body length after 20 min in the presence of 500 µM of three phenolamines: OA, TA (the precursor to OA) and synephrine (SE, the methylated product of OA). We also tested the structurally related catecholamines, dopamine (DA), norepinephrine (NE) and metanephrine (ME), and three other BAs, namely, 5HT, histamine (HA) and phenylethylamine (PE; Fig. 4). DA signaling has been more extensively studied in schistosomes (Pax et al., 1984; Taman and Ribeiro, 2009; El-Shehabi et al., 2012) and any phenotypic changes measured here can be compared with previous studies, as described below.

**Fig. 5.**
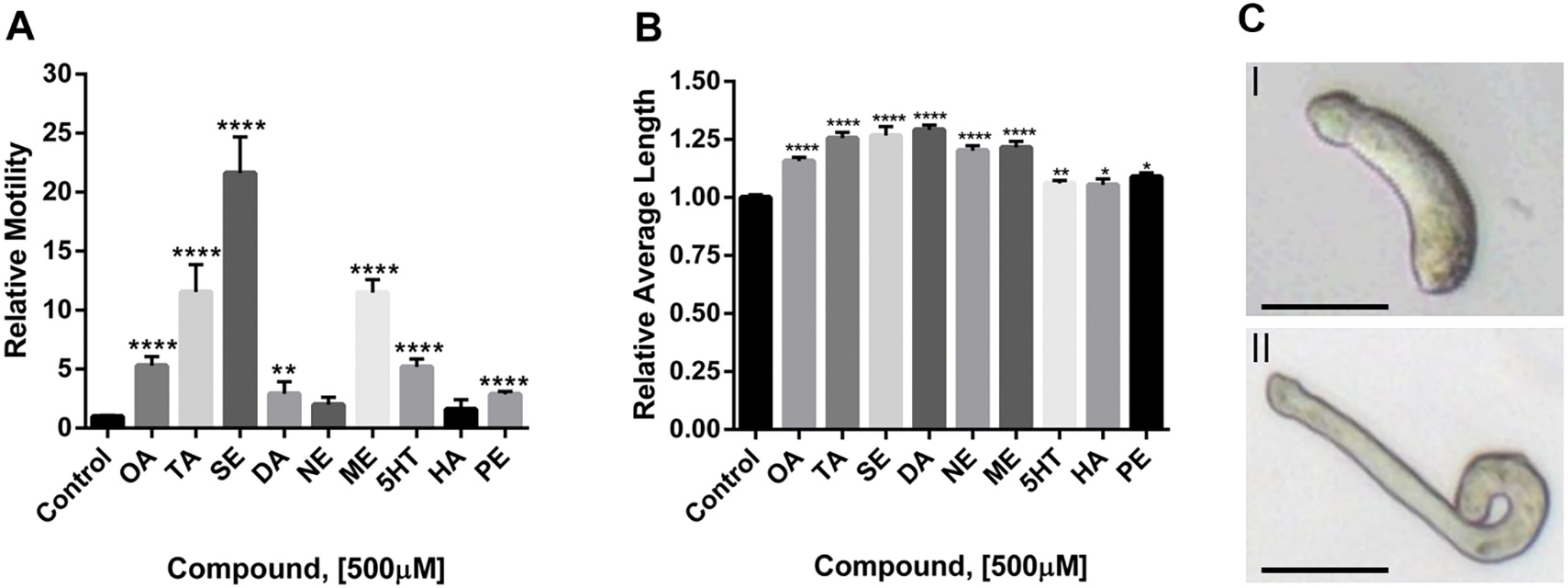
**BAs increase motility and body length.** D7 schistosomula were incubated with BAs at a concentration of 500 µM for 20 min. The BAs tested were octopamine (OA), tyramine (TA), synephrine (SE), dopamine (DA), noradrenaline (NE), metanephrine (ME), serotonin (5HT), histamine (HA) and phenylethylamine (PE). (A) Relative motility and (B) length were quantified as described in the text and are expressed as the fold change compared with schistosomula in the presence of 0.015% DMSO for SE or H_2_O for all other compounds. The mean relative motility and length were measured by normalization to each well, at baseline. Means and s.e.m. from a minimum of two experiments with a minimum of two replicates per experiment are shown (*n*=36). Significance of the mean values, compared with controls, was determined using the unpaired two-tailed Student's *t*-test: *P*-values <0.05*, <0.005** and <0.00005**** were considered significant. (C) Representative images of (I) a control and (II) an OA-treated parasite: note the length of the parasite exposed to OA relative to control. Scale bars: 50 µm.

All three phenolamines significantly increased schistosomulum motility over the water or DMSO controls (Fig. 5A). The strongest stimulation (∼22-fold) was observed with SE, followed by TA (12-fold) and then OA (5-fold). For the catecholamines, the strongest effect on motility was observed with ME (14-fold) followed by DA and NE (2-3-fold; the latter non-significantly). For the other BAs, 5HT increased motility 5-fold, a value similar to the 3-fold increase noted previously for *S. mansoni* schistosomula (Patocka et al., 2014) and sporocysts (Boyle and Yoshino, 2005) that parasitize the snail vector host. HA was inactive, in contrast to the 50% increase in motility noted previously for cercariae (Ercoli et al., 1985), and PE increased motility 5-fold.

In terms of average length, all three phenolamines, OA, TA and SE, generated a trend similar to that regarding motility with significant increases of approximately 20-25% (Fig. 5B,C). Likewise, the catecholamines DA, NE and ME increased the average length of schistosomula by approximately 25%. DA, at 0.5 µM, has previously been shown to lengthen the body of adult parasites by up to 20% after 3 min (Tomosky et al., 1974). Finally, the other BAs tested, 5HT, HA and PE, generated a smaller, yet statistically significant 10% increase in average body length consistent with their less dramatic effects on motility.

**Fig. 6.**
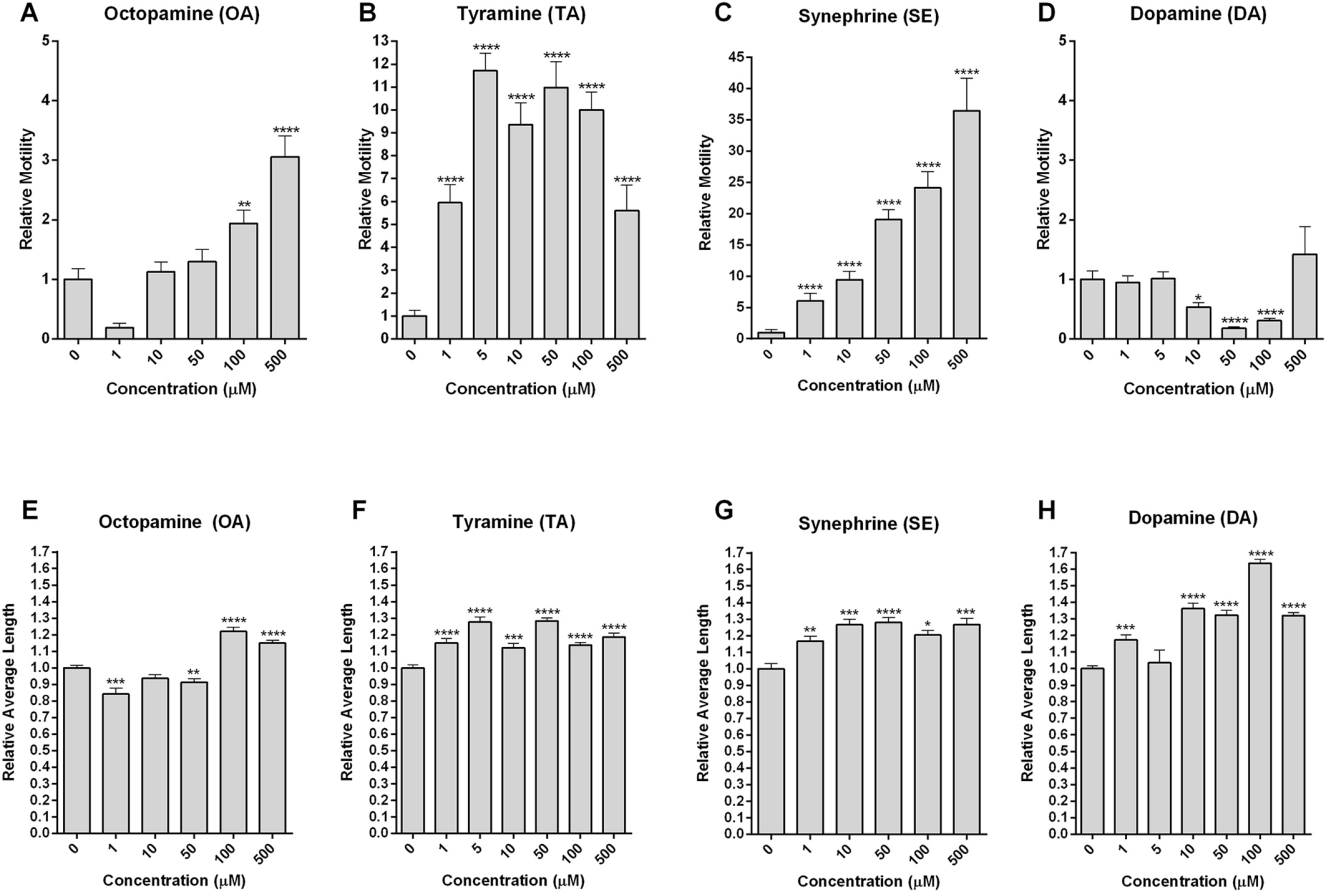
**OA and related amines modulate motility and length of schistosomula.** Using D7 schistosomula, concentration-response assays (1-500 μM) were performed over 20 min with four tyrosine derivatives previously determined to cause significant changes in motility at 500 μM (Fig. 5). The mean relative motility (A-D) and length (E-H) were measured by normalization to each well, at baseline. Means and s.e.m. from a minimum of two experiments with a minimum of two replicates per experiment are shown (*n*=36). The data presented at the 500 µM concentration are distinct from the data presented in Fig. 5. Significance of the mean values, compared with controls, was determined using the unpaired two-tailed Student's *t*-test: *P*-values <0.05*, <0.005**, <0.0005*** and <0.00005**** were considered significant.

We then performed concentration-response assays (1-500 µM) for motility (Fig. 6A-D) and length (Fig. 6E-H) with the three phenolamines, OA, TA and SE, and the catecholamine DA. OA and SE both demonstrated linear increases in kinetics for motility and, to a smaller degree, worm length. The methylated phenolamine SE was the most powerful agent (i.e. a 25-fold increase in motility at 100 µM versus 2-fold for OA). By contrast, a biphasic response that was intermediate between OA and SE was recorded for the OA precursor, TA: motility increased by a maximum of approximately 10-fold between 5 and 100 µM. Unlike the phenolamines, DA induced a biphasic concentration-dependent inhibition of motility (≥50% reduction in motility relative to control) that was maximal between 10 and 100 µM. This finding is consistent with a previous demonstration that DA causes a significant decrease in motility in schistosomula at concentrations from 10 to 100 µM over 5 min (El-Shehabi et al., 2012). Like the phenolamines, which increased motility and length (Fig. 6A-C,E-G), the decreased motility induced by DA at 100 µM was also associated with an increase in length by an impressive 65% over control.

### Drug agonists and antagonists of tyrosine-derivative signaling influence schistosome motility and length

We performed a phenotypic screen of D7 schistosomula with 28 drug modulators of tyrosine-derivative signaling to identify possible starting points for new drugs (Table 1). The compounds were selected from a collection of diverse small molecules maintained at the University of California, San Francisco (UCSF) Small Molecule Discovery Center. Compounds were tested at a concentration of 65 µM (i.e. in the middle of the 1-500 µM range already tested with the BAs).

**Table 1.**
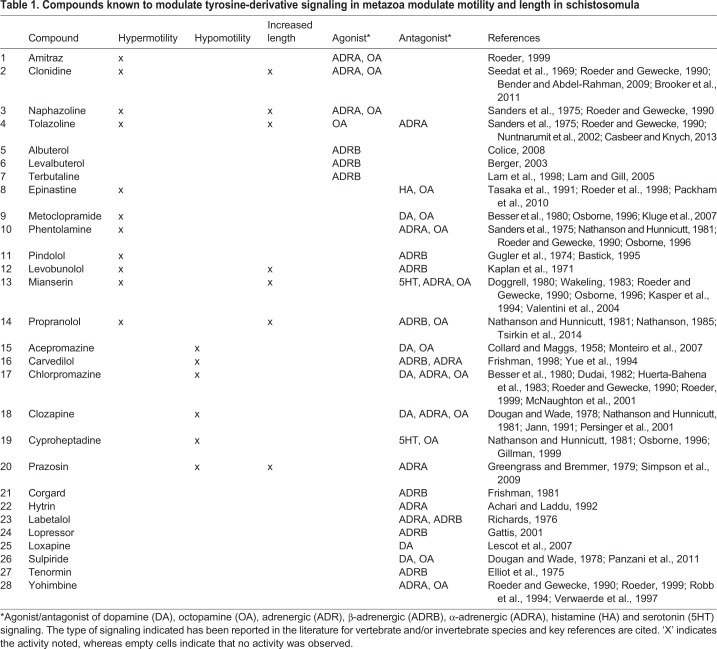
**Compounds known to modulate tyrosine-derivative signaling in metazoa modulate motility and length in schistosomula**

Hypermotility, with or without parasite lengthening, could be induced by drugs that agonize or antagonize OA signalling; for example, amitraz and epinastine agonize and antagonize OA receptors, respectively, in mites, cattle ticks and locusts (Evans and Gee, 1980; Hollingworth and Murdock, 1980; Dudai, 1982; Roeder et al., 1998). Modulators of adrenergic signaling, clonidine, naphazoline, tolazoline and propranolol (PR), also increased motility and length (Table 1). Given the homology between OA and adrenergic receptors, and the similarity in their pharmacological profiles (Evans, 1981, 1985; Roeder, 1995, 1999; Blenau et al., 2012), these drugs might act on the schistosome OA system.

Hypomotility was more often associated with drugs that antagonize OA and adrenergic receptors; that is, acepromazine, carvedilol (CAR), chlorpromazine (CPZ), clozapine, cyproheptadine and prazosin (Table 1 and Movies 5-8). Among these, prazosin was unique in that, in addition to inducing hypomotility, it increased worm length. The hypomotile effect of cyproheptadine could be attributable to an antagonistic effect on OA signaling, as has been shown to occur in locust motor control (Evans, 1981). Of the 28 compounds tested, 11 did not alter schistosomulum motility or length. This might be due to a lack of penetration of the parasite during the 20 min assay time and/or insufficient engagement of the relevant receptor(s). Nine of the 11 drugs are reported to act on adrenergic receptors, of which six specifically target the β-receptor subtype. This finding might suggest that there are more β- than α-adrenergic-like receptors in the parasite.

**Fig. 7.**
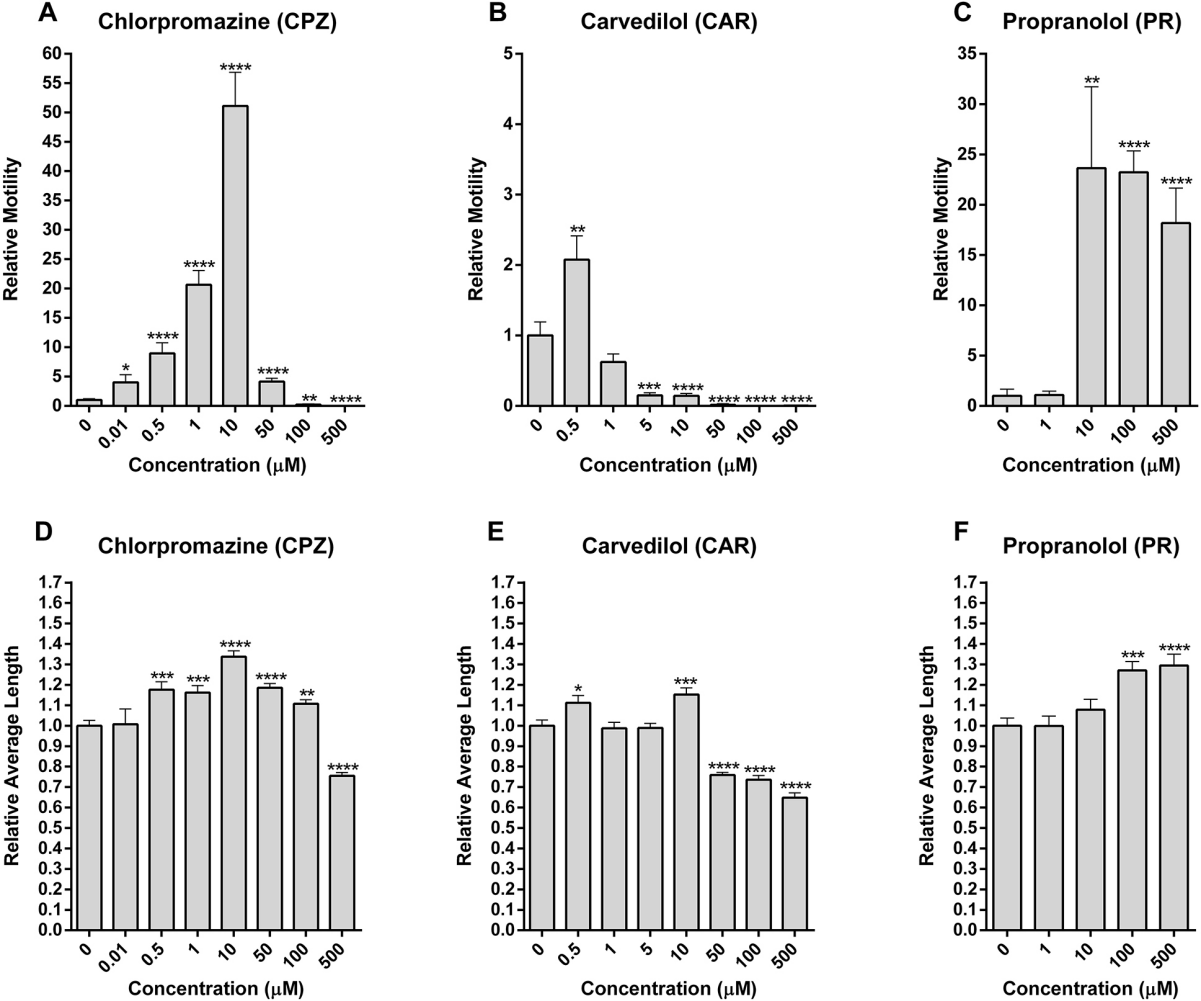
**Chlorpromazine, carvedilol and propranolol modulate motility and length of schistosomula.** Using D7 schistosomula, concentration-response assays (0.01-500 μM) were performed over 20 min for three modulators of tyrosine-derivative signaling that had caused pronounced effects on (A-C) motility and (D-F) length at 65 μM (Table 1). Mean relative motility values were determined by normalization to the corresponding wells at baseline (preceding addition of the drug compound; *n*≥13). Means and s.e.m. from a minimum of three experiments, each with a minimum of two replicates per experiment, are shown. A minimum of three wells were recorded per treatment. Note that there is no continuity of scale in the *y*-axis between graphs A-C. Significance of the mean values, compared with controls, was determined using the unpaired two-tailed Student's *t*-test: *P*-values <0.05*, <0.005**, 0.0005*** and <0.00005**** were considered significant.

Three adrenergic antagonists, CPZ, CAR and PR (Fig. 7), were selected for concentration-response assays (1-500 µM) owing to their pronounced effects on motility and length. CPZ is also known to antagonize the *S. mansoni* DA receptor, SmD2 (Taman and Ribeiro, 2009). As stated above, vertebrate adrenergic antagonists/agonists might also modulate OA signaling in the schistosome.

The three drugs elicited varying and complex responses in the parasite (Fig. 7). The effect of CPZ on motility and length was biphasic (Fig. 7A,D). Motility increased up to a maximum of 50-fold over that of control at 10 µM and was followed by paralysis at 100 µM and 500 µM. The data are consistent with previous reports demonstrating enhanced motility of 1-day-old schistosomula after 2 h in the presence of 1 µM CPZ (Abdulla et al., 2009) and over-activity in 2-day-old schistosomula in the presence of 5 µM CPZ (Weeks et al., 2018). CPZ also increased body length up to a maximum of 30% over control at 10 µM, with a subsequent decline to 30% below control at 500 µM. In a recent report (Weeks et al., 2018), increased length was also recorded for schistosomula at low concentrations (5 µM), whereas shortening was observed at a higher concentration (10 µM). The biphasic responses recorded could indicate saturation of one receptor type at ever-increasing concentrations of drug or might suggest that more than one receptor type is being engaged, which would be consistent with CPZ's action on both adrenergic and DA receptors. The paralysis recorded at 100 µM and above might be due to spasticity resulting from a continued stimulation of muscle contraction (Blair et al., 1988). Stimulation of muscle contraction through antagonism could result from antagonism of receptors that are associated with muscle relaxation.

CAR was essentially a paralytic across most of the concentrations tested with a 20-30% shortening of the body at higher concentrations (50-500 µM; Fig. 7B,E). These effects on schistosomula are opposite to those in the presence of the natural phenolamines, OA, TA and SE (increased motility and length; Fig. 6), and might indicate that CAR indeed operates as an OA antagonist in schistosomes.

Finally, PR increased motility, particularly between 1 and 10 µM, with a sustained maximum increase of 17- to 24-fold above 10 µM that was associated with a 30% increase in length (Fig. 7C,F). Accordingly, PR might operate as an OA agonist in the schistosome, contrary to its known activity as a weak antagonist in insects such as locusts (Evans, 1981) and crickets (Stevenson et al., 2005).

## DISCUSSION

### Motor control as a drug target in parasitic helminths

Disruption of neuromuscular control is a successful anthelmintic strategy. The current anti-schistosomal PZQ putatively engages a β-subunit of calcium channels (Jeziorski and Greenberg, 2006), causing an influx of calcium and tetanic paralysis (Andrews et al., 1983). The older anti-schistosomal drug, metrifonate, inhibits acetylcholinesterase resulting in flaccid paralysis (Bueding et al., 1972; Day et al., 1994). In parasitic nematodes, ivermectin binds glutamate-gated chloride channels inducing an influx of calcium and subsequent flaccid paralysis (Campbell and Blair, 1978; Cully et al., 1994).

BAs typically signal through G-protein-coupled receptors (GPCRs), however, the current knowledge of BA GPCRs in schistosomes is fragmented (Hahnel et al., 2018). DA, HA and 5HT GPCRs have been characterized in *S. mansoni* (Taman and Ribeiro, 2009; El-Shehabi and Ribeiro, 2010; El-Shehabi et al., 2012; Patocka et al., 2014). Also, two putative GPCRs are annotated in the schistosome genome as OA or TA GPCRs (Smp_150180 and Smp_043290; Protasio et al., 2012), although these receptors have not yet been characterized.

Most studies on BA signaling in schistosomes have focused on 5HT (Catto and Ottesen, 1979; Pax et al., 1984; Patocka and Ribeiro, 2013; Patocka et al., 2014) with fewer reports on the roles of the catecholamine DA (Pax et al., 1984; El-Shehabi et al., 2012). However, the contribution of phenolamines like OA to schistosome motor control is unknown. Understanding and disrupting this signaling system offers an inherently attractive anthelmintic prospect given its absence from vertebrates (Degen et al., 2000). The mapping and phenotypic studies reported here represent a first step in this direction and already highlight the often complex response modalities of the schistosome to drugs (Fig. 7), which, elsewhere, are reported to antagonize OA/adrenergic signaling.

### The organization of the nervous system in the parasitic life stages of *S. mansoni* and the discovery of two pairs of ganglia in the brain

Previous studies involving electron microscopy (Cousin and Dorsey, 1991) and confocal immunolabeling (Skuce et al., 1990; Mair et al., 2000; Halton and Maule, 2004; Collins et al., 2011; McVeigh et al., 2011) have shown that the trematode nervous system possesses a pair of CG and three pairs of longitudinal NCs (ventral, lateral and dorsal). Moreover, the longitudinal NCs are cross-linked by transverse commissures along the length of the worm generating an orthogonal (ladder-like) pattern (Hyman, 1951; Halton and Maule, 2004). For adult *S. mansoni* specifically, we demonstrate this orthogonal pattern using an anti-synapsin antibody that had been previously used to map the nervous system in *S. mansoni* cercariae (Collins et al., 2011).

Importantly, through the use of both synapsin- and OA-specific antibodies, we discover two pairs of ganglia (AG and PG) in the adult schistosome brain. Although there are reports of a single CG pair in different developmental stages of the parasite (Cousin and Dorsey, 1991; Gustafsson, 1987; McVeigh et al., 2012), this is the first time that two pairs of ganglia have been identified in the brain in a single schistosome stage using a neuronal marker (Fig. 2A,B, Fig. S1). A double bi-lobed structure has been described for *Paricterotaenia* spp. tapeworms (Kotikova and Raikova, 2008). Tapeworm species have brains at various levels of evolutionary development: a ‘simple’ brain with little organization, a ‘circular’ ganglionic structure, a brain with two clearly formed lobes, and the most advanced four-lobed brain (Kotikova and Raikova, 2008).

The presence of one ganglia pair next to each of the ventral and oral suckers in the brain of the mature schistosome suggests a particular adaption to both the independent and coordinated control of each sucker as the animal moves through the mesenteric blood system, feeds through the oral sucker and finds a mate. Based on previous findings of a single CG pair anterior to the ventral sucker in the cercariae (Cousin and Dorsey, 1991) and our own findings here with schistosomula, it seems that one pair of ganglia appears first with the second pair developing as the parasite grows and matures in the mammalian host. Apart from the two pairs of ganglia, the synapsin antibody clearly labels the rest of the CNS (i.e. the longitudinal NCs and transverse commissures) in the adult schistosome and schistosomula. We also observed a web-like pattern of synapsin-labeling in the embryo.

Having confirmed the utility of synapsin as a neuronal marker in various schistosome life stages, we attempted to localize OA, the presence of which had not yet been determined for this parasite. OA was localized in both pairs of ganglia, specifically in the cell bodies along the ganglia surfaces. OA was also distributed throughout the CNS and PNS. In the adult male, the close juxtaposition of OA and synapsin in the tegumental tubercles suggests a function for OA in sensation and efferent signaling. Furthermore, the presence of OA in a nerve fiber running along the parasite's caecum might indicate a function in gut peristalsis. Finally, OA was found in neurons lining the female reproductive tract, including the ovary, and in neurons of the developing embryo, suggesting a role for OA in egg production and development. A contribution of OA to egg laying has been reported in the nematode *Caenorhabditis* *elegans* (Horvitz et al., 1982; Alkema et al., 2005; Chase and Koelle, 2007).

### Tyrosine derivatives modulate schistosome motor control

Given the widespread distribution of OA, including in the peripheral nerve plexus of *S. mansoni* (Movie 4) that typically innervates body wall muscle (Halton and Maule, 2004), OA might be important for schistosome motility. To explore this possible function, we incubated D7 schistosomula with OA, six related tyrosine derivatives and two other common BAs (5HT and HA), and measured changes in motility and length using an established quantitative assay (El-Shehabi et al., 2012; Patocka and Ribeiro, 2013; Patocka et al., 2014; Macdonald et al., 2015). Apart from the availability of an assay to determine motility, schistosomula can be derived in large numbers (thousands of larvae from the snail hosts) without the need for a mammalian host, as would otherwise be the case when working with adult parasites (Abdulla et al., 2009).

The phenolamines OA, TA and SE increased schistosomular motility in a concentration-dependent manner with maximal increases of 3-, 12- and 35-fold, respectively. The more pronounced effect of SE on motility is consistent with a trend observed previously for methylated derivatives of 5HT and DA (El-Shehabi et al., 2012; Patocka et al., 2014). Also, in the primary screen of the catecholamines performed here, it could be noted that incubation with the methylated ME was more myo-stimulatory than either DA or NE. The finding for SE, with its extra methyl group, might suggest that it has a higher affinity for OA receptors and/or the extra methyl group enhances uptake and delivery to target receptors by virtue of its greater lipophilicity.

Unlike OA and SE, biphasic responses were measured for TA and the catecholamine DA. These results highlight the complexity of neuromuscular control, such that a continuum of target proteins or groups of proteins might be engaged as a function of concentration to alter the overall motor phenotype. This complexity is augmented by the presence of three muscle layers, circular, longitudinal and diagonal, in the parasite (Mair et al., 2000).

OA, TA and SE also increased schistosomular length in a concentration-dependent manner (up to 30%), and the impressive 60% increase in length induced by DA is consistent with previous reports for both schistosomula and adult schistosomes (Pax et al., 1984; El-Shehabi et al., 2012). These increases in length might result from a relaxation of longitudinal muscle and/or a contraction of circular muscle (Pax et al., 1984).

### Drugs that act on tyrosine-derivative signaling modulate schistosome motor control

Having demonstrated that phenolamines and catecholamines alter schistosome shape and motility, and with a view to identifying new drugs for schistosomiasis, we phenotypically screened 28 drug agonists and/or antagonists of tyrosine-derivative signaling for changes in length and motility (Table 1). We included drugs that interfere with adrenergic signaling and that might also act on the OA system, given the homology between OA and adrenergic receptors and their similar pharmacological profiles (Evans, 1981, 1985; Roeder, 1995, 1999; Blenau et al., 2012), while accepting that inconsistencies in agonistic/antagonistic effects can exist between vertebrates and invertebrates, and even within invertebrates (Osborne, 1996). We also tested drugs that modulate DA signaling (Besser et al., 1980; Gillman, 1999; Panzani et al., 2011).

Marked phenotypic changes were observed for many of the drugs tested: some compounds induced hypermotility with or without increases in length, whereas others decreased motility. One drug, the adrenergic antagonist prazosin, decreased parasite length. Nonetheless, some trends were noted. Hypomotility was more associated with antagonistic drugs, whereas hypermotility, with or without parasite lengthening, could be induced by either agonistic or antagonistic drugs. Also, those drugs reported to more specifically act on β-adrenergic receptors did not alter length or motility, which might suggest that the putative OA receptors engaged could be more α- than β-adrenergic-like.

From the phenotypic screen, the adrenergic antagonists and anti-hypertensive drugs CAR (Frishman, 1998) and PR (Ramirez and Talmers, 1985; Tsirkin et al., 2014), respectively, were selected for concentration-response studies. Both drugs might alter OA signaling in schistosomes, based on findings that modulators of adrenergic signaling in vertebrates also affect phenolamine signaling in invertebrates (Dougan and Wade, 1978; Roeder and Gewecke, 1990). The induction of pronounced paralysis and decreased parasite length by CAR is consistent with its known adrenergic antagonism. By contrast, the increase in both motility and length by PR would suggest that the drug acts as an agonist in the schistosome, contrary to its previously described antagonistic behavior (Mcneill, 1964; Nathanson and Hunnicutt, 1981). Thus, further investigation is warranted to understand the mechanism by which PR acts on the parasite.

CPZ, a mixed adrenergic and DA antagonist (Masri et al., 2008), was the third drug chosen for concentration-dependent studies. CPZ potently antagonizes the *S. mansoni* DA receptor, SmD2, in a heterologous expression system when tested at concentrations ranging from 0.1 to 100 µM (Taman and Ribeiro, 2009); that is, comparable to the 0.01 to 500 µM range tested here. The biphasic response noted for both worm motility and length might indicate saturation of one receptor type at ever-increasing concentrations of drug or a more complicated situation that involves more than one receptor type, perhaps across different muscle layers.

The marked dysregulation of the parasite's neuromuscular control by the drugs tested, including those tested at low micromolar concentrations, is encouraging from a drug repositioning standpoint. In humans, CPZ and CAR attain peak plasma concentrations of approximately 0.158 µM (Kolakowska et al., 1976) and 41.82 µM (Kindermann et al., 2004), after therapeutic doses of up to 100 and 25 mg/day, with half-lives of 11 h (Yeung et al., 1993) and 7-10 h (Stafylas and Sarafidis, 2008), respectively. In our 20 min quantitative assay, CPZ and CAR caused hypomotility and hypermotility at 0.01 µM and 0.5 µM, respectively, well within the plasma concentration and time envelopes. PR, by contrast, only reaches a peak plasma concentration of approximately 0.054 µM (Paterson et al., 1970) after a therapeutic dose of up to 40 mg/day, well below the concentration necessary (1-10 µM) to induce phenotypic changes *in vitro*. Furthermore, in mice, for which a well-established *S. mansoni* infection model is available, the tolerated doses for CPZ, CAR and PR are 10 mg/kg (Petruska et al., 2002), 200 mg/kg (Food and Drug Administration Coreg Data Sheet; https://www.accessdata.fda.gov/drugsatfda_docs/label/2015/020297s037lbl.pdf) and 20 mg/kg (Ristori et al., 2011), respectively. In line with the current standard of drug care for schistosomiasis, single doses would be administered such that any long-term effects of treatment, as can occur with adrenergic antagonists like CAR (Berglund and Andersson, 1981) and CPZ (Keks, 1996; Tada et al., 2004), would be less of a concern.

Apart from the possibility of repositioning current drugs, a future chemical optimization program would involve identifying the relevant target(s), and then improving binding specificity and potency. As already noted, the schistosome DA receptor, SmD2, has already been expressed and shown to be antagonized by CPZ (Taman and Ribeiro, 2009). RNA interference might also provide insight into the contributions of specific receptors in motor control, as was the case for serotonergic receptors in schistosomula and adult parasites (Patocka et al., 2014) and acetylcholinergic receptors in schistosomula (Macdonald et al., 2015).

To conclude, using an antibody specific to synapsin, we describe the organization of the nervous system of those schistosome life-stages that are parasitic in the mammalian host; unique to trematodes so far, we have revealed the presence of two pairs of ganglia in the schistosome brain. We also identify the presence and wide distribution of OA, an invertebrate-specific signaling molecule. Finally, we measured the effects on parasite length and motility of natural BAs and synthetic agonists and antagonists of tyrosine-derivative signaling. A number of drugs that might be useful in the development of specific schistosomicides were identified.

## MATERIALS AND METHODS

### Parasites

*S. mansoni*-infected *Biomphalaria glabrata* snails were supplied by the Schistosomiasis Resource Center at the Biomedical Research Institute (BRI, Rockville, MD, USA). To obtain schistosomula, infected snails were induced to ‘shed’ cercariae (infective larvae) under light exposure for 2 h. Cercariae were collected and allowed to settle at 4°C for 1 h. They were then resuspended in Dulbecco's Modified Eagle Medium (DMEM; Thermo Fisher Scientific, MA) containing 500 μg/ml streptomycin and 500 U/ml penicillin (Gibco, Thermo Fisher Scientific, MA) and mechanically transformed to schistosomula by vortexing (Lewis et al., 1986). Schistosomula were cultured in a humidified incubator at 37°C and 5% CO_2_.

To obtain adult parasites, 28-day-old female CD1 mice were each infected with ∼200 cercariae by immersing the tail in water containing the parasite (Smithers and Terry, 1965; Tucker et al., 2013). Mice were sacrificed after 7-8 weeks and adult worms collected by perfusion of the mesenteric and the hepatic portal veins (Smithers and Terry, 1965; Carneiro and Lopes, 1986; Lewis, 2001).

All vertebrate animal procedures were approved under Protocol Number 3346 by McGill University's Macdonald Campus Facility Animal Care Committee, and are in keeping with the guidelines of the Canadian Council on Animal Care.

### Confocal microscopy

Parasites were washed, permeabilized and fixed for confocal immunolocalization as described (Mair et al., 2000; Taman and Ribeiro, 2009; Patocka et al., 2014) with some modifications. Freshly collected adult worms were washed in Opti-MEM^TM^ (Thermo Fisher Scientific) and incubated at room temperature for 25 min in a 6-well plate to promote separation of paired males and females. Adult worms were then washed five times in phosphate-buffered saline (PBS), placed between two glass slides and submerged in 4% paraformaldehyde (PFA) at 4°C for 4 h. Schistosomula were cultured for 7 days, washed twice in PBS and incubated in 4% PFA for 4 h with end-over-end rotation.

Following fixation, worms were washed three times in PBS, followed by a 5 min wash in 100 mM glycine in PBS. Worms were permeabilized in 2% SDS for 2 h and then washed three times in antibody diluent (AbD; PBS containing 0.5% Triton-X-100 and 0.1% BSA) followed by an overnight incubation at 4°C with end-over-end rotation. Worms were subsequently probed with primary antibody specific for the protein or BA of interest in AbD, individually or in combination. Negative controls included parallel experiments omitting primary antibody and, for the anti-OA antibody, pre-adsorption with 0.19 g/ml (1 mM) OA.

The primary antibodies, namely, anti-OA (EMD Millipore, Merck Millipore), anti-5HT (EMD Millipore, Merck Millipore) and a monoclonal anti-synapsin antibody (anti SYNORF1, submitted to the Developmental Studies Hybridoma Bank, DSHB; http://dshb.biology.uiowa.edu/) were added at dilutions of 1:80, 1:100 and 1:35, respectively, and incubated for 3 days at 4°C with end-over-end rotation. For schistosomula, anti-synapsin antibody was added at a titer of 1:25 rather than 1:35. Adults and schistosomula were washed three times and once, respectively, and incubated overnight in AbD.

Secondary antibody was added at a dilution of 1:800 in an overnight incubation. In some experiments, 4′,6-diamidino-2-phenylindole dihydrochloride (DAPI; Thermo Fisher Scientific) at 1:500 (20 ng/ml) was also added to stain nuclei. Incubations, with or without DAPI, were then maintained for 2 days. The secondary antibodies used included an Alexa-488-conjugated goat anti-rabbit antibody (Thermo Fisher Scientific) and a horse radish peroxidase (HRP)-conjugated goat anti-mouse antibody (Santa Cruz Biotechnology, TX). As a negative control, adults and schistosomula were incubated without the primary synapsin antibody and then washed four times and twice, respectively, in AbD before mounting for confocal microscopy.

Worms probed with anti-synapsin were prepared as follows. Following incubation with secondary antibody, the Alexa-594-conjugated tyramide signal amplification reagent (TSA™ Kit #15, Thermo Fisher Scientific) was added to the AbD, supplemented with 0.0015% H_2_O_2_, at a dilution of 1:80 and incubated at 4°C for 1 h with end-over-end rotation. Adult worms were washed five times, and schistosomula three times, before mounting. Mounted specimens were visualized using a Zeiss LSM710 confocal microscope (Carl Zeiss Inc., Oberkochen, Germany) operated by the ZEN 2010 software (Carl Zeiss Inc.). Argon (488 nm) and helium-neon (594 nm) lasers were used to excite dyes and fluorophores and to obtain images. Filter sets were adjusted to minimize overlap of emission wavelengths and non-specific bleed-through of the acquired signal owing to spectral overlap. A complete list of antibodies and reagents used in this study is provided in Table S1.

### Phenotypic screening of schistosomula with BAs

*In vitro*-transformed schistosomula were cultured in 24-well plates at ∼150 units/well for 7 days at 37°C in 850 μl Opti-MEM^TM^ supplemented with 5% heat-inactivated FBS, 100 μg/ml streptomycin and 100 U/ml penicillin. Animals were acclimated to room temperature for 15 min and motility was analyzed to give baseline readings using a previously described protocol (El-Shehabi et al., 2012; Patocka and Ribeiro, 2013; Patocka et al., 2014; Macdonald et al., 2015). The test compound was administered at concentrations ranging from 0 (vehicle alone control) to 500 μM. Recordings were taken before the addition of compound and 20 min following the addition of compound or vehicle (H_2_O or 0.015% DMSO). Schistosomula were recorded for 1 min with a Nikon SMZ1500 microscope equipped with a QICAM Fast 1394 (mono 12-bit, QImaging, CA) digital video camera and video acquired with SimplePCI software (version 5.2, Compix Inc., OR) at ∼2.5 frames/s (fps). A minimum of 12 schistosomula were recorded per experiment across a minimum of three distinct fields of view from two to three wells per treatment. To avoid bias in selection, all schistosomula in the field of the video were analyzed, unless in contact with one another. ImageJ (version 1.41, NIH, MD) Fit Ellipse algorithm was used to quantify worm motility by measuring the changes in worm length over 60 s, as described (Patocka and Ribeiro, 2013). The software was used to determine the length of the major axis along the ellipse of best fit in each frame of video, as an indicator of changes in length. Motility and length measurements were expressed as a fold change over the mean of each well at baseline.

### Phenotypic screening of schistosomula with drug agonists and antagonists of tyrosine-derivative signaling

Screens were performed with schistosomula prepared using agonists and antagonists of phenolamine and catecholamine signaling selected from a small-molecule collection that contains FDA-approved drugs (Pharmakon Pharmaceuticals, Inc., IN). Preference was given to compounds that affect OA signaling in invertebrates and adrenergic signaling in vertebrates (Table 1). Compounds that modulate adrenergic signaling were selected as OA signaling can perform several of the functions of adrenergic signaling in invertebrates (Evans et al., 1976; Evans, 1981) and because compounds that modulate adrenergic signaling can inhibit OA and TA receptors with similar potencies (Evans, 1981; Roeder, 1999; Blenau et al., 2012).

Cercariae were obtained from *B. glabrata* snails, suspended in Basch medium (Basch, 1981) and transformed to schistosomula by passage through a 22 G double-headed Luer Lock needle to remove cercarial tails (Colley and Wikel, 1974; Abdulla et al., 2009; Štefanić et al., 2010). Schistosomula were washed over ice three times in medium supplemented with 80 μg/ml streptomycin and 80 U/ml penicillin. The parasites were resuspended in Basch medium supplemented with 2.5% FBS and added to 96-well round-bottomed clear plastic plates. Images of schistosomula were acquired in time-lapse in an IN Cell Analyzer 2000 (GE Healthcare, IL) over 20 s at 1.66 fps. Parasite motility and length in the presence and absence of compound were compared and assessed manually by playing the 20 s recordings (generated using ImageMagick; http://www.imagemagick.org/) side-by-side. Videos were also compared with wells to which vehicle was added and assessed manually. All experiments were performed in triplicate and a minimum of 15 animals per well were analyzed.

### Statistical analysis

*P*-values for the two-tailed Student's *t-*tests were calculated in Excel^®^ 2007 (Microsoft Corporation, WA, USA) using the function: TTEST(array1, array2, 2, 2) that compares treated (array 1) and control (array 2) raw motility and length values.

## Supplementary Material

Supplementary information
