# Supplementary material for: Octopamine signaling in the metazoan pathogen Schistosoma mansoni: localization, small-molecule screening and opportunities for drug development
Source: Dis Model Mech. 2018 Jul 30;11(7):dmm033563. doi: 10.1242/dmm.033563 (PMC6078403; doi:10.1242/dmm.033563)
Supplement: Supplementary information [file dmm-11-033563-s1.pdf]

FIRST PERSON

First person – Nelly El-Sakkary

First Person is a series of interviews with the first authors of a selection of papers published in Disease Models & Mechanisms, helping early-career researchers promote themselves alongside their papers. Nelly El-Sakkary is first author on 'Octopamine-signaling in the metazoan pathogen *Schistosoma mansoni*: localization, small-molecule screening and opportunities for drug development', published in DMM. Nelly conducted the research while a PhD student in the lab of Paula Ribeiro at McGill University, Canada. She is now a postdoctoral researcher in Conor R. Caffrey's lab at the University of California, San Diego, USA, where she focuses on improving human health through pre-clinical drug discovery using molecular and genomic tools.

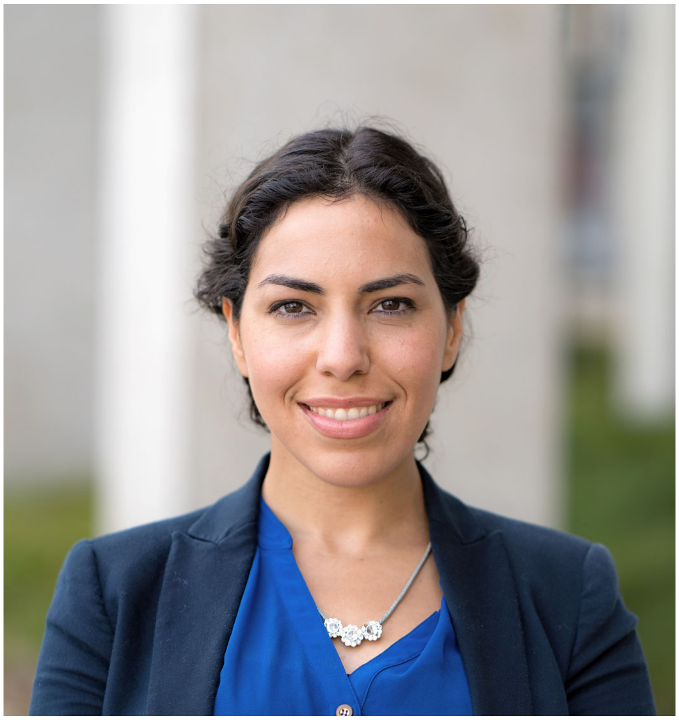

Nelly El-Sakkary

How would you explain the main findings of your paper to non-scientific family and friends?

We discovered that the parasitic flatworm, *Schistosoma*, has a neurotransmitter, octopamine, which plays an important role in controlling its movement. We tested different neurotransmitters and modulators of these neurotransmitter signaling pathways on parasites. Octopamine and related neurotransmitters all caused a pronounced increase in movement. We also used laser microscopy to map the nervous system, localize octopamine throughout the nervous system and to determine that the flatworm brain is made up of four distinct lobes, rather than two lobes as was previously reported.

What are the potential implications of these results for your field of research?

Schistosoma is a globally important, yet understudied pathogen. There is only one drug used in its treatment, praziquantel, raising concerns for the emergence of resistance. It is important to seek out new drug targets and develop novel treatments for the disease. Other successful anthelmintics like ivermectin and levamisole demonstrate that interfering with worm motor control is a viable treatment that does not require killing the parasite, but allows for clearance from the host. The importance of octopamine in *Schistosoma* motor control, therefore, suggests strong potential for drug design to treat schistosomiasis.

What are the main advantages and drawbacks of the model system you have used as it relates to the disease you are investigating?

The disadvantage of this model system is that generating flatworms takes much time and effort: the life cycle requires a snail and rodent host to generate the flatworm and typically takes three months. However, the advantage of working with the flatworms is that it allows direct testing of compounds on the parasites and observation of schistosome neurobiology. These aspects have measurable outputs, including detecting motor control changes and neuronal localization.

Nelly El-Sakkary's contact details: Center for Discovery and Innovation in Parasitic Diseases, Skaggs School of Pharmacy and Pharmaceutical Sciences, University of California, 9500 Gilman Drive, La Jolla, CA 92093, USA.
E-mail: nelsakkary@ucsd.edu

What has surprised you the most while conducting your research?

What surprised me the most was the findings of the confocal immunolocalization studies. Namely, the four lobes in the brain and the abundance of octopamine throughout the nervous system. These findings suggest that octopamine is an important schistosome neurotransmitter and that the brain is, perhaps, more complex than previously thought.

> "[…] it is primarily a disease of the developing world, […] which means, unfortunately, that the field is underfunded and understudied."

Describe what you think is the most significant challenge impacting your research at this time and how will this be addressed over the next 10 years?

The most significant challenge impacting my research is the attention given to the field of neglected tropical disease (NTD) research, including schistosomiasis. These diseases are 'neglected', in large part, because there is a lack of resources dedicated to NTD research. Despite schistosomes infecting hundreds of millions of people globally, it is primarily a disease of the developing world, as are other NTDs, which means, unfortunately, that the field is underfunded and understudied. Despite this neglect, advances in NTDs continue to be made, in large part due to funding from both governmental and non-profit organizations. For this manuscript we received funding from Canadian, American and British funding agencies, including

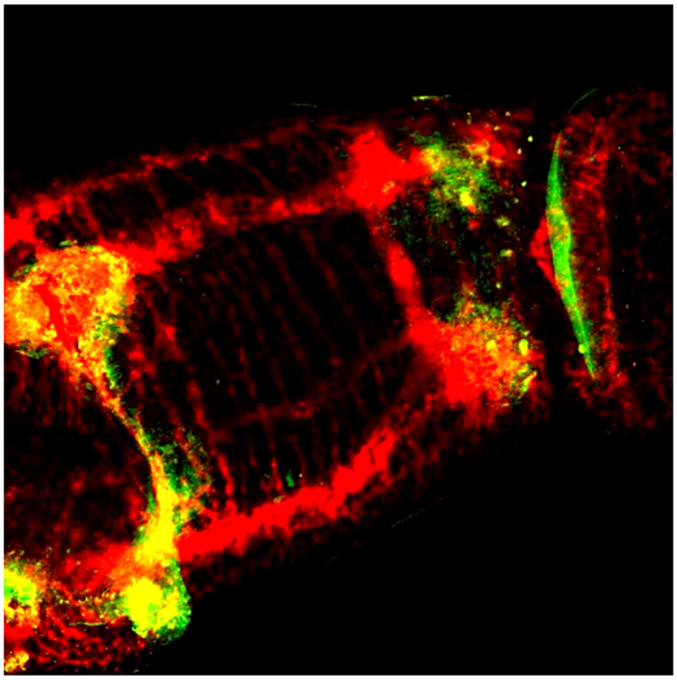

Adult worm brain showing octopamine (green) and synapsin (red). Regions of overlap are indicated in yellow, demonstrating octopamine in the lobes of the brain. There are four lobes, two anterior ganglia (right), and two posterior ganglia (left) making up the brain, rather than two lobes as previously reported.

The Company of Biologists, who helped make this research possible. In the next 10 years, NTD research must continue to receive financial support, to continue making advancements in understanding parasite biology and in drug discovery.

What changes do you think could improve the professional lives of early-career scientists?

Changes in academia that can help improve the professional lives of early-career scientists include introducing more structure into a scientist's training. Much of the learning process is informal and varies greatly depending on the lab environment and the trainee's supervisor/s. Standardizing the training process nationally, for both supervisor and trainee, in the form of courses, goal-setting and check-points could help to facilitate the training/learning process and ensure success.

What's next for you?

My plans are to complete my postdoc in the chemical and gene-expression profiling of the schistosome parasite at UC San Diego and to seek employment. I want to continue performing pre-clinical drug discovery to develop tools to treat globally important diseases. I am also interested in translational research for the betterment of human and animal health.

Reference

El-Sakkary, N., Chen, S., Arkin, M. R., Caffrey, C. R. and Ribeiro, P. (2018). Octopamine-signaling in the metazoan pathogen *Schistosoma mansoni*: localization, small-molecule screening and opportunities for drug development. *Dis. Model. Mech.* **11**: dmm033563.
